# Daytime, Not Nighttime, Elevated Atmospheric Carbon Dioxide Exposure Improves Plant Growth and Leaf Quality of Mulberry (*Morus alba* L.) Seedlings

**DOI:** 10.3389/fpls.2020.609031

**Published:** 2021-02-04

**Authors:** Songmei Shi, Yuling Qiu, Miao Wen, Xiao Xu, Xingshui Dong, Chenyang Xu, Xinhua He

**Affiliations:** ^1^Centre of Excellence for Soil Biology, School of Resource and Environment, Southwest University, Chongqing, China; ^2^Key Laboratory of Southwest China Wildlife Resources Conservation (China West Normal University), Ministry of Education, Nanchong, China; ^3^School of Biological Sciences, University of Western Australia, Perth, WA, Australia

**Keywords:** biomass production, CO_2_ enrichment, free amino acid, mineral elements, nutrient use efficiency

## Abstract

Almost all elevated atmospheric CO_2_ concentrations (eCO_2_) studies have not addressed the potential responses of plant growth to different CO_2_ in daytime and nighttime. The present study was to determine the impact of daytime and/or nighttime eCO_2_ on growth and quality of mulberry (*Morus alba* L.), a perennial multipurpose cash plant. Six-month-old mulberry seedlings were hence grown in environmentally auto-controlled growth chambers under four CO_2_ concentrations: (1) ambient CO_2_ (ACO_2_, 410 μmol mol^–1^ daytime/460 μmol mol^–1^ nighttime), (2) sole daytime elevated CO_2_ (DeCO_2_, 710 μmol mol^–1^/460 μmol mol^–1^), (3) sole nighttime elevated CO_2_ (NeCO_2_, 410 μmol mol^–1^/760 μmol mol^–1^), and (4) continuous daytime and nighttime elevated CO_2_ (D + NeCO_2_, 710 μmol mol^–1^/760 μmol mol^–1^). Plant growth characteristics, nutrient uptake, and leaf quality were then examined after 120 days of CO_2_ exposure. Compared to control, DeCO_2_ and (D + N)eCO_2_ increased plant biomass production and thus the harvest of nutrients and accumulation of leaf carbohydrates (starch, soluble sugar, and fatty acid) and N-containing compounds (free amino acid and protein), though there were some decreases in the concentration of leaf N, P, Mg, Fe, and Zn. NeCO_2_ had no significant effects on leaf yield but an extent positive effect on leaf nutritional quality due to their concentration increase in leaf B, Cu, starch, and soluble sugar. Meanwhile, (D + N)eCO_2_ decreased mulberry leaf yield and harvest of nutritious compounds for silkworm when compared with DeCO_2_. The reason may be associated to N, P, Mg, Fe, and Zn that are closely related to leaf pigment and N metabolism. Therefore, the rational application of mineral nutrient (especially N, P, Fe, Mg, and Zn) fertilizers is important for a sustainable mulberry production under future atmosphere CO_2_ concentrations.

## Introduction

The increasing atmosphere CO_2_ concentration has become one of the worldwide hot issues. The atmosphere CO_2_ concentration has increased from 280 μmol mol^–1^ in the pre-industry times to 417 μmol mol^–1^ in November 2020^1^ and is predicted to exceed 700 μmol mol^–1^ by the end of the twenty-first century (IPCC,, 2014). As atmospheric CO_2_ is the primary source of carbon (C) for plants, the ongoing increased CO_2_ will act as a C fertilizer, resulting in an increase in biomass production in cereal crops (Drag et al., 2020; Park et al., 2020; Tcherkez et al., 2020), vegetable crops (Dong et al., 2020), perennial fruit crops (Salazar-Parra et al., 2015), grass (Xiao et al., 2016), and some perennial woody plants (Maillard et al., 2001; Singh et al., 2019; Ahammed et al., 2020). The beneficial effect has led to a growing demand for macro- and micronutrients, including nitrogen (N), phosphorous (P), potassium (K), calcium (Ca), magnesium (Mg), etc., to match their increased C assimilation under elevated CO_2_ (eCO_2_), resulting in changes in crop quality and hence food nutrition (Loladze, 2014; Kohler et al., 2019; Chumley and Hewlings, 2020). Therefore, to address how eCO_2_ affects crop productivity, food quality and security is timely needed.

Mulberry (*Morus alba* L.) is a fast-growing multipurpose plant, and its leaves are rich in proteins, carbohydrates, fats, fibers, minerals, and vitamins (Butt et al., 2008). In a number of Asian and European countries, mulberry leaves have been used not only for rearing silkworm (*Bombyx mori* L.) but also for feeding cattle, goat, and other animals, being used as tea and vegetable, and treating atherosclerosis, diabetes mellitus, etc. (Papanastasis et al., 2008; Guha et al., 2010). The quality of mulberry leaves thus strongly associates with the quantity and quality of cocoon, animal, and human nutrition. However, little information was available about the responses of nutritional quality in mulberry leaves under global environmental change scenarios. To date, only limited reports have explored the response of mulberry trees to eCO_2_. For example, Sekhar et al. (2014, 2015) showed that plant height, leaf numbers, branches, total shoot length, and biomass production in 6 month-old mulberry Selection-13 (S13) and Kanva-2 (K2) genotypes were lower under ambient CO_2_ than under 550 μmol mol^–1^ eCO_2_ for 90 days. In addition, such a 550 μmol mol^–1^ eCO_2_ treatment significantly increased net photosynthetic rates, intercellular CO_2_ concentration, photosynthetic N and water use efficiency, and Rubisco, chlorophyll a, starch, and total sugar concentrations, but significantly decreased stomatal conductance, transpiration rates, and light compensation point in the fully expanded upper third or fourth leaf, respectively (Sekhar et al., 2015). Meanwhile, all abovementioned increases were higher under S13 than under K2 (Sekhar et al., 2015). Recently, 800 μmol mol^–1^ eCO_2_ alleviated drought stresses on 1 year “Qinglong” mulberry seedlings by increasing their leaf water use efficiency and PSII photochemical activity (Liu et al., 2019). The number and biomass of inflorescence in female, not in male of the mulberry tree, were increased after 18 months eCO_2_ (ambient plus 380 μmol mol^–1^ CO_2_ exposure) (Li et al., 2019). Nevertheless, relatively less attention has been paid to their changes into leaf nutrition quality of mulberry trees, especially their macro- and micronutrients under eCO_2_, considering that N, P, K, Ca, Mg, boron (B), zinc (Zn), iron (Fe), copper (Cu), and manganese (Mn) in the mulberry leaves are essential to silkworm and other animals (Radojkovic et al., 2014).

A 7 year experiment of free-air CO_2_ enrichment (FACE) study reported that N, P, and Zn concentrations on grain were, respectively, decreased by 6, 5, and 10% under 550 eCO_2_, irrespective of soil types, crop species, and year (Jin et al., 2019). Moreover, short-term FACE studies showed that 550–900 μmol mol^–1^ eCO_2_ decreased P in *Medicago truncatula* (Jakobsen et al., 2016), Ca, Zn, and Mn in lettuce (Baslam et al., 2012), and oilseed rape (Högy et al., 2010), and Zn and Fe in grains of wheat (*Triticum aestivum*) (Fernando et al., 2012), and soybean (*Glycine max*) (Kohler et al., 2019). The adverse effect of 550–900 μmol mol^–1^ eCO_2_ on leaf and grain quality was also observed with decreased economic benefits of food crops (Dietterich et al., 2015). However, all of these studies have determined the responses of plant growth and nutrient uptake to eCO_2_ only at daytime, but not at nighttime.

The atmospheric CO_2_ concentrations indeed differ between daytime and nighttime, considering that differences exist in plant photosynthesis and soil respiration particularly in agricultural fields. For example, a 3 month (June–September, assuming in 1999 or 2000) observation in Australia, Japan, and United States showed that the average atmospheric CO_2_ concentration at canopy height (rice and other crops/weeds) varied from 390 μmol mol^–1^ at daytime to 465 μmol mol^–1^ at nighttime (Ziska et al., 2001). A few studies have indicated that eCO_2_ at nighttime can influence plant growth and dry matter accumulation, and the positive or negative effects on growth result from effects of CO_2_ on dark respiration (Bunce, 1995, 2002; Griffin et al., 1999). However, there are controversial effects of eCO_2_ at nighttime on dark respiration. It is suggested that 500–1,400 μmol mol^–1^ eCO_2_ at nighttime decreased both respiration and translocation processes in short-term experiments, and the responses of common bean (*Phaseolus vulgaris*) growth depended on whether CO_2_ was elevated at nighttime or daytime (Amthor et al., 1992; Bunce, 2002). In contrast, dark respiration usually, but not always, increases in proportion to the elevated photosynthesis rate in long-term CO_2_ enrichment experiments (Poorter et al., 1992; Bunce, 1995). Mechanisms by which eCO_2_ at night may influence plant growth have not been established, and their effect on plant carbon balance and nutrient allocation also has not been revealed. Thus, variations in daytime and/or nighttime eCO_2_ concentrations shall provide a closer simulation of future atmospheric CO_2_ conditions that plants will respond to in the near future.

Several methodologies have been explored to simulate the response of a plant–soil system to eCO_2_. For example, closed-chamber experiments can strictly control environmental factors, e.g., temperature, humidity, water, light, CO_2_ concentration, etc., which affect plant growth in a completely closed space (Freijer and Bouten, 1991). This closed-chamber system has been widely applied in agricultural research, although the environments are often substantially different from those in the field (Horie et al., 1995; Cheng et al., 2006). The open-top chamber (OTC) is a semi-closed climate simulation system with all chamber sides being closed but the top opened. The OTC is comparatively inexpensive, but has a higher air temperature and humidity, lower solar radiation and wind turbulence, thus decreasing evapo-transpiration and increasing pest and disease (Lawlor and Mitchell, 1991; Amthor, 2001). Compared to the conventional closed-chamber and OTC, the free-air carbon dioxide enrichment (FACE) creates a microclimate to approach the natural environment; however, it is hard to achieve a high CO_2_ enrichment during nighttime because of the extra expense or the lack of wind at night to deliver the CO_2_ to the plots (Norby and Zak, 2011; Kuzyakov et al., 2019). By taking into account the advantages and disadvantages of these systems, in the present study, we constructed an automatically environmentally controlled glass-made growth chambers that was programmed to mimic outside environmental conditions except for CO_2_ concentration, which was set to the amounts required for the experiment during daytime and nighttime (Supplementary Figure S1). With comparisons to the current CO_2_ atmosphere concentrations, the objectives of the present study were to address how daytime and/or nighttime eCO_2_ could affect: (1) mulberry biomass production, (2) leaf concentrations of macronutrients (N, P, K, Ca, and Mg) and micro-nutrients (Mn, B, Cu, Fe, and Zn), and (3) leaf concentrations of carbohydrates (starch, soluble sugar, and fatty acid) and N-containing compounds (free amino acid and protein). Six month-old mulberry seedlings were therefore grown inside environmentally controlled glass-made chambers, which had the same growth conditions (fertilization, temperature, humidity, etc.), except CO_2_ concentrations: ambient CO_2_, eCO_2_ in daytime only, eCO_2_ in nighttime only, and continuous eCO_2_ in both daytime and nighttime. The abovementioned plant characteristics were then compared after a further 120 day growth.

## Materials and Methods

### Plant Materials and Experimental Design

The seeds of mulberry (*Morus alba* var. Gui-sang-you 62) were provided by the Sericultural Research Institute of Sichuan Academy of Agricultural Sciences, and the test materials were 6 month older mulberry seedlings with uniform growth status. One seedling was planted in a plastic pot (20 × 24 cm = height × diameter) filled with 5 kg of soil (Eutric Regosol, FAO Soil Classification System). The soil (pH 6.8) had 7.56 g of organic carbonkg^–1^, 0.66 g of total Nkg^–1^, 0.61 g of total Pkg^–1^, 97 mg available Nkg^–1^, 17 mg available Pkg^–1^, and 197 mg available K kg^–1^.

**FIGURE 1:**
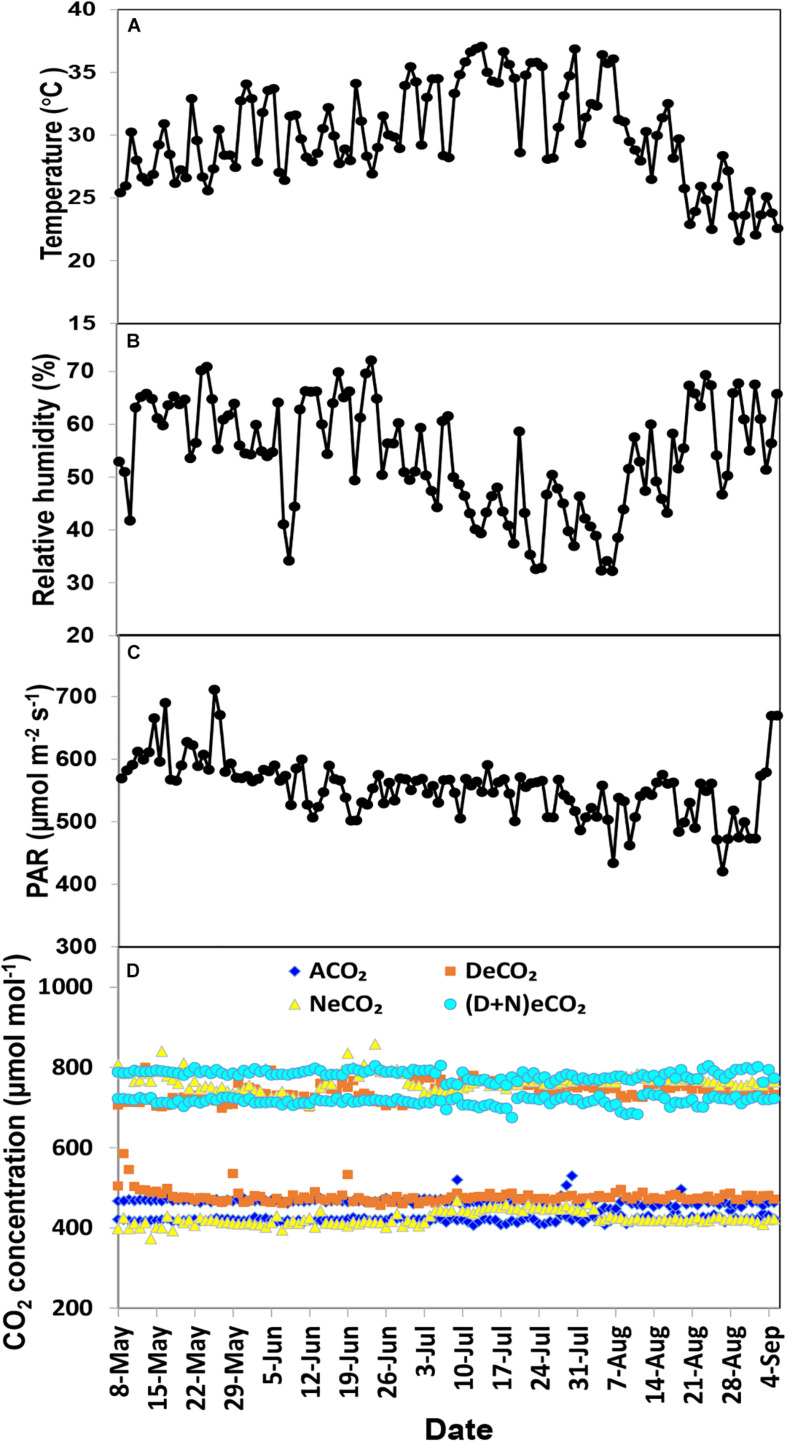
Mean temperature **(A)**, relative humidity **(B)**, photosynthetic active radiation (PAR) **(C)**, and CO_2_ concentration **(D)** over the experimental period in the growth chambers.

A total of 12 glass-made growth chambers (length × width × height = 1.5 × 1.0 × 2.5 m) were constructed in the National Monitoring Base for Purple Soil Fertility and Fertilizer Efficiency (29°48′N, 106°24′E, 266.3 m above sea level) on the campus of Southwest University, Chongqing, China. The four-side walls and top roof of the chamber were constructed using tempered glasses (10 mm thickness) with 90% light transmission rate (Yutao Glass Company, Jiulongpo, Chongqing 400051, China). The CO_2_ concentration, temperature, and humidity inside the growth chambers were auto-controlled by a CO_2_ auto-controlling facility (DSS-QZD, Qingdao Shengsen Research Institute of CNC Technology, Shandong, China). Based on the on-site variation of daytime and nighttime atmosphere CO_2_ concentrations, we designed four CO_2_ treatments: (1) ambient CO_2_ (ACO_2_, 410/460 μmol mol^–1^ nighttime), (2) elevated CO_2_ in daytime only (DeCO_2_, 710/460 μmol mol^–1^), (3) elevated CO_2_ in nighttime (NeCO_2_, 410/760 μmol mol^–1^), and (4) elevated CO_2_ in both daytime and nighttime [(D + N)eCO_2_, 710/760 μmol mol^–1^]. Daytime was from 07:30 a.m. to 19:30 p.m. and nighttime was from 19:30 p.m. to 07:30 a.m. Each CO_2_ treatment had three independent replicates or chambers, and each chamber contained six independent mulberry seedlings (pots) for a total of 18 seedlings. Except for CO_2_ concentrations, those growth chambers had the same growth conditions including fertilization, light, temperature, and humidity. The temperature and humidity inside and outside the growth chambers were kept consistent with each other by an auto-controlling facility. The photosynthetic active radiation (PAR) was supplied by the natural light, though the tempered glass had 90% light transmission. The variations in mean daily temperature, relative humidity, PAR, and CO_2_ concentration over the whole growth stage are shown in Figure 1. The pots in the chambers were weekly relocated once in order to ensure a similar plant growth environment.

### Plant Sampling and Measurements

After 120 days of CO_2_ exposure, plant samples were harvested and divided into leaf, stem, and root, and their fresh weight was then, respectively, measured. The harvested fresh leaves were divided into two groups. One group of the fresh leaves was cut into fine pieces and mixed for the determination of soluble sugar, starch, and free amino acid, and the measured data were then calculated on the basis of their dry weight. Another group of fresh leaves, all harvested fresh stems, and roots were oven dried at 75°C until their dry weight was constant. The oven-dried leaf, stem, or root samples were ground into fine powder for the determination of crude fatty acid (leaf only) or element concentrations. The determined elements were C, N, P, K, Ca, Mg, Mn, B, Cu, Fe, and Zn for leaf samples, but only C for stem and root samples that were presented in this study. The total plant biomass was the sum of leaf, stem, and root.

The concentrations of C in plant tissues were determined with the potassium dichromate–sulfuric acid oxidation method, and the concentrations of leaf N was determined with the Kjeldahl method to determine protein levels (N × 6.25) (Yang et al., 2008). After the nitric acid digestion, the mineral elements, including P, K, Ca, Mg, Fe, Zn, B, Cu, and Mn, were determined by using an inductively coupled plasma-optical emission spectrometry (iCAP 6500 Duo; Thermo Fisher Scientific, Waltham, MA, United States), following the operation procedures in the spectrometry’s manual.

Determination of leaf soluble sugar (mg g^–1^ DW) and starch (mg g^–1^ DW) was followed by the anthrone method using glucose as the standard (Li, 2000). Briefly, 200 mg of fresh leaves were extracted with 10 ml of ethanol (80%, v/v) in a water bath at 80°C for 30 min, and centrifuged at 13,000 rpm for 10 min. The supernatant was collected into a 100 ml volumetric flask for estimating soluble sugar. The residue after the final centrifugation was added with 5 ml of H_2_O and 2 ml of perchloric acid (9.2 mol L^–1^), and then centrifuged at 13,000 rpm for 10 min. The supernatant was collected into a 100 ml volumetric flask for estimating starch. One milliliter of supernatant from both samples was added with 5 ml of freshly prepared anthrone sulfuric acid solution (80%, v/v), respectively, and incubated in boiling water for 10 min. After cooling, the absorbance of the incubated supernatant was spectrophotometrically read at 620 nm.

Determination of free amino acids (mg g^–1^ DW) was performed by ninhydrin colorimetry using leucine as standard (Li, 2000). Briefly, 500 mg of fresh leaves was extracted with 10 ml of H_2_O in boiling water for 20 min, and then the mixture was centrifuged at 13,000 rpm for 10 min. The supernatant was collected into a 100 ml volumetric flask. One milliliter of the extraction was added into 0.5 ml of NaCN (0.01 mol L^–1^) and 0.5 ml ninhydrin (3%, w/v), and the mixture was boiled for 12 min. After cooling, 5 ml of ethanol (95%, v/v) was added into the mixture, and the absorbance of the incubated supernatant was spectrophotometrically read at 570 nm.

Determination of leaf crude fatty acid (mg g^–1^ DW) was followed by the Soxhlet extractor method (Gao, 2006). Briefly, 20,000 g of dried leaf powder was placed in a filter cartridge, which was dried at 105°C for 2 h, then the cartridge was added with 50 ml of anhydrous ether, and refluxed for 12 h using a Soxhlet apparatus. After the anhydrous ether was fully evaporated, the cartridge was oven dried at 105°C for 2 h, cooled in a desiccator, and then weighed.

Leaf nutrient accumulation was calculated by the leaf concentration and biomass. Leaf nutrient accumulation (mg plant^–1^) = leaf biomass (g plant^–1^) × leaf nutrient concentration (mg g^–1^). The nutrient use efficiency was calculated according to Carvalho et al. (2020): Nutrient use efficiency (g) = (leaf biomass, g plant^–1^)^2^ / (nutrient accumulation, mg plant^–1^).

### Statistics

Data (means ± SE, *n* = 3) were statistically analyzed by one-way ANOVA with SPSS 19.0. Significant differences between treatments were analyzed by the Duncan’s multiple range test at *P* < 0.05 using the SPSS 19.0 (SPSS Inc., Chicago, IL, United States). The polynomial regression analysis was performed using the OriginPro 8.0 (OriginLab Corp., Northampton, MA, United States).

## Results

### Plant Biomass Production

**FIGURE 2:**
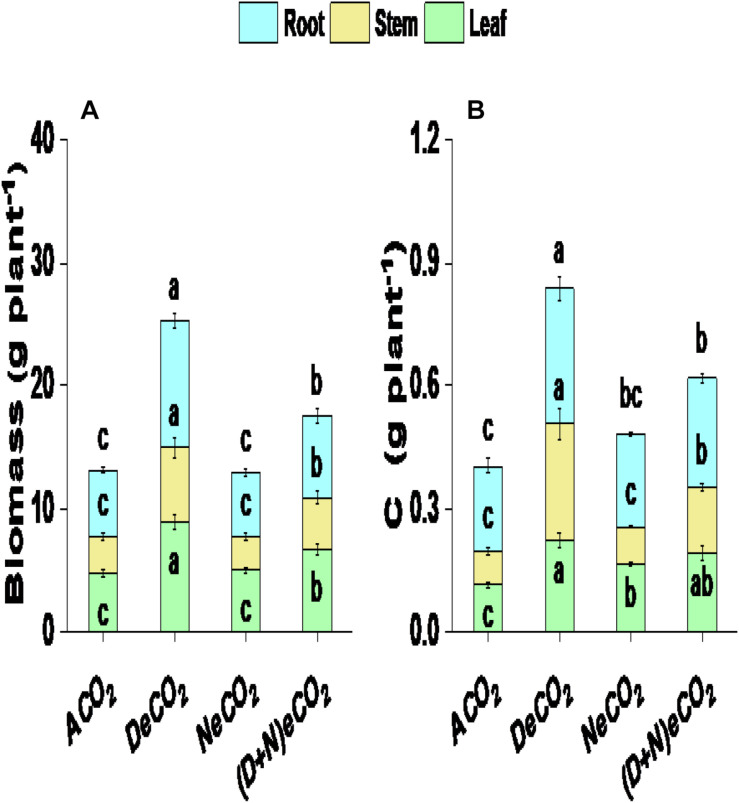
Plant biomass production **(A)** and carbon accumulation **(B)** of mulberry seedlings grown for 120 days under four different daytime and nighttime CO_2_ concentrations inside environmentally controlled glass growth chambers. Data (means ± SE, *n* = 3) followed by different letters above the bars are significant differences between CO_2_ treatments at *P* < 0.05. Abbreviations: ACO_2_, ambient CO_2_ (410 μmol mol^–1^ daytime + 460 μmol mol^–1^ nighttime); DeCO_2_, sole daytime elevated CO_2_ (710 μmol mol^–1^ daytime + 460 μmol mol^–1^ nighttime); NeCO_2_, sole nighttime eCO_2_ (410 μmol mol^–1^ daytime + 760 μmol mol^–1^ nighttime); (D + N)eCO_2_, continuous daytime/nighttime eCO_2_ (710 μmol mol^–1^ daytime + 760 μmol mol^–1^ nighttime). All CO_2_ concentrations had a variation of ± 30 μmol mol^–1^ in inside environmentally controlled growth chambers. Daytime: 07:30 a.m.–19:30 p.m. and nighttime: 19:30 p.m.–07:30 a.m.

The 710/460 μmol mol^–1^ DeCO_2_ significantly enhanced leaf biomass by 102%, stem biomass by 187%, root biomass by 90%, and total plant biomass by 113%, compared with mulberry plants grown under ACO_2_ (Figure 2A). Leaf, stem, root, and total plant biomass production in mulberry grown under 710/760 μmol mol^–1^ (D + N)eCO_2_ was, respectively, increased by 42, 42, 22, and 40% compared with their ambient counterparts (Figure 2A). However, 410/760 μmol mol^–1^ NeCO_2_ had no significant effects on plant biomass production, including leaf, stem, and root biomass (Figure 2A). Significantly higher leaf, stem, root, and total plant biomass between CO_2_ treatments is ranked as DeCO_2_ > (D + N)eCO_2_ > NeCO_2_ ≈ ACO_2_ (Figure 2A)_._

### Carbon Accumulation

DeCO_2_ and (D + N)eCO_2_ treatments significantly increased C accumulation in leaf, stem, and root by 68–95, 94–245, and 27–58%, respectively (Figure 2B). Leaf and root C accumulation under NeCO_2_ was, respectively, increased by 45 and 10%, whereas stem C basically had no changes, compared with mulberry plants grown under ACO_2_ (Figure 2B). Between CO_2_ treatments, significantly higher C accumulation is ranked as DeCO_2_ > (D + N)eCO_2_ > NeCO_2_ > ACO_2_ for leaf and root and DeCO_2_ > (D + N)eCO_2_ > NeCO_2_ ≈ ACO_2_ for stem (Figure 2B).

### Leaf Concentrations and Accumulations of Mineral Elements

**FIGURE 3:**
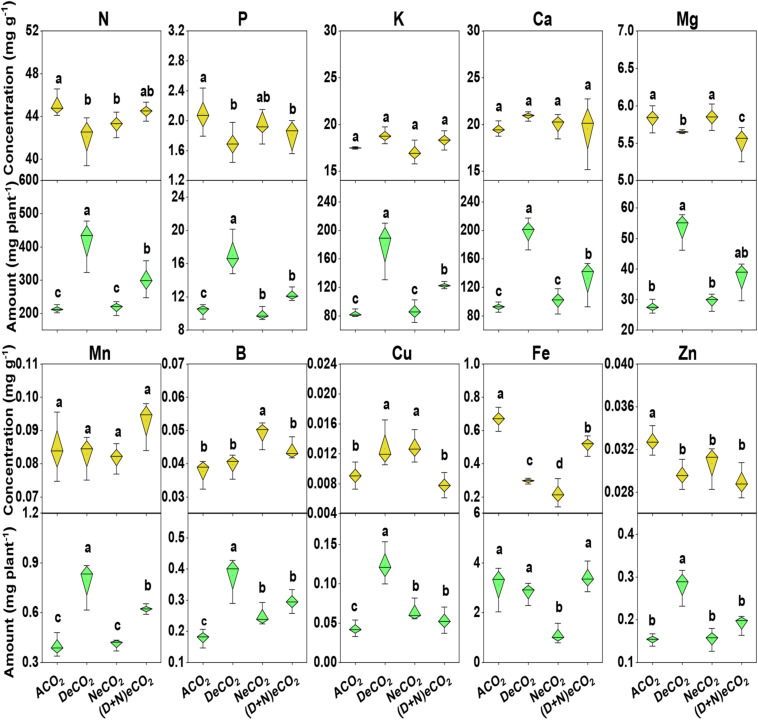
Concentrations and accumulations of leaf macro–micro elements in mulberry seedlings grown for 120 days under four different daytime and nighttime CO_2_ concentrations inside environmentally controlled glass growth chambers. Different letters indicate significant differences among CO_2_ treatments at *P* < 0.05. See treatment abbreviations in Figure 1.

Irrespective of CO_2_ treatments, concentrations of leaf K, Ca, Mn, and B were generally similar, except for a higher B or Cu under NeCO_2_ or DeCO_2_ (Figure 3). Compared to ACO_2_, leaf N, P, Mg, Fe, and Zn concentrations declined by 5.0–6.0, 9–18, 3.0–5.3, 24–67, and 6.3–11.7% under eCO_2_ enrichment, respectively. Among the four CO_2_ treatments, significantly higher nutrient concentration is ranked as ACO_2_ ≈ (D + N)eCO_2_ > NeCO_2_ ≈ DeCO_2_ for leaf N, ACO_2_ > NeCO_2_ > (D + N)eCO_2_ ≈ DeCO_2_ for leaf P, ACO_2_ ≈ NeCO_2_ > DeCO_2_ > (D + N)eCO_2_ for Mg, ACO_2_ > (D + N)eCO_2_ > DeCO_2_ > NeCO_2_ for Fe, and ACO_2_ > NeCO_2_ ≈ DeCO_2_ ≈ (D + N)eCO_2_ for Zn (Figure 3).

Compared with ACO_2_, DeCO_2_ significantly increased accumulations of leaf N, P, K, Ca, Mg, Mn, B, Cu, and Zn, but did not change Fe accumulation (Figure 3); (D + N)eCO_2_ significantly increased plant N, P, K, Ca, Mg, Mn, B, and Cu accumulations, but had a similar leaf Fe and Zn accumulations (Figure 3). NeCO_2_ had significantly higher leaf B and Cu, lower leaf Fe accumulation, but no effects on leaf N, P, K, Ca, Mg, Mn, and Zn accumulations, compared with these under ACO_2_ (Figure 3).

### Nutrient Use Efficiency

**FIGURE 4:**
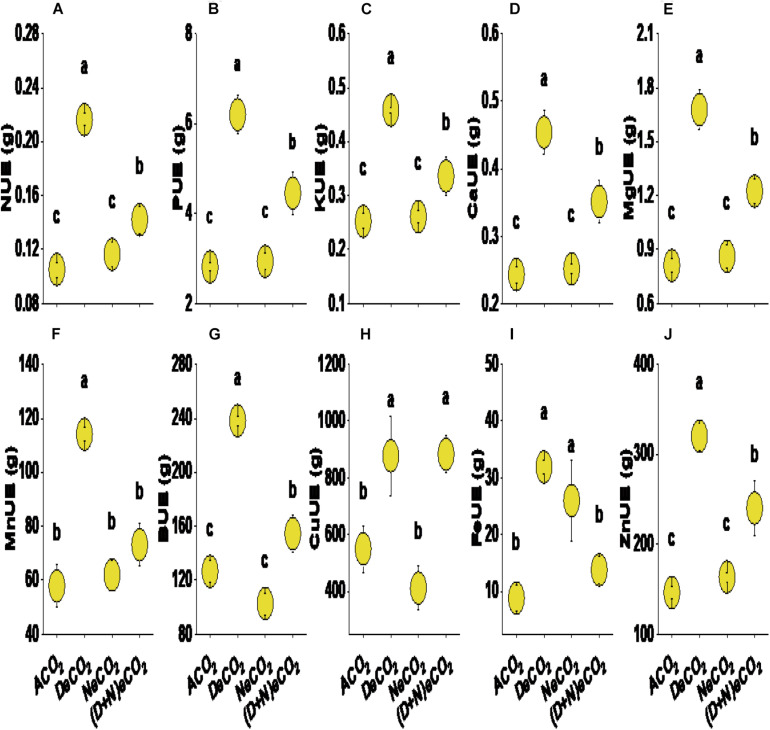
Nitrogen (N, **A**), phosphorus (P, **B**), potassium (K, **C**), calcium (Ca, **D**), magnesium (Mg, **E**), manganese (Mn, **F**), boron (B, **G**), copper (Cu, **H**), zinc (Zn, **I**) and iron (Fe, **J**) use efficiency in the leaves of mulberry seedlings grown for 120 days under different daytime and nighttime CO_2_ concentrations inside environmentally controlled glass growth chambers. Different letters indicate significant differences among CO_2_ treatments at *P* < 0.05. See treatment abbreviations in Figure 1; UE, use efficiency.

Compared with ACO_2_, both DeCO_2_ and (D + N)eCO_2_ significantly increased the nutrient use efficiency of N, P, K, Ca, Mg, Mn, B, Cu, and Fe. Both DeCO_2_ and NeCO_2_ significantly increased the nutrient use efficiency of Fe. Nutrient use efficiency was highest in the elevated CO_2_ at daytime (increased by 60–260% compared with ACO_2_), followed by continuous elevation of CO_2_ (22–64% increase, compared with ACO_2_), while no effects were detected under eCO_2_ at nighttime, except for nutrient use efficiency of Fe (Figure 4).

### Leaf Quality Variables

**FIGURE 5:**
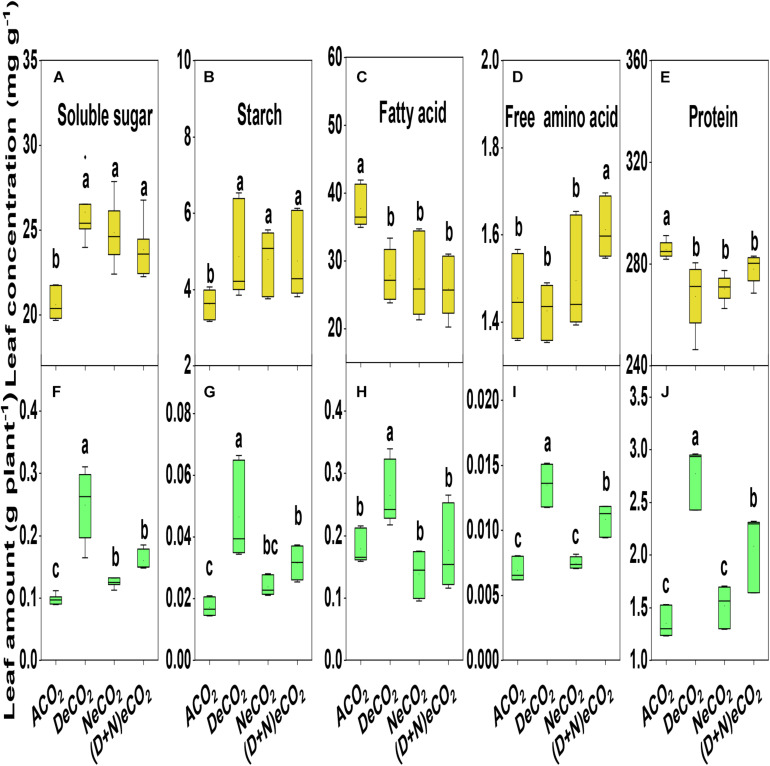
Concentrations and accumulations of leaf soluble sugar **(A,F)**, starch **(B,G)**, fatty acid **(C,H)**, total free amino acid **(D,I)**, and protein **(E,J)** of mulberry seedlings grown for 120 days under different daytime and nighttime CO_2_ concentrations inside environmentally controlled glass growth chambers. Different letters indicate significant differencesamong CO_2_ treatments at *P* < 0.05.

eCO_2_ significantly increased the concentrations of leaf soluble sugar and starch by 13–28 and 31–35%, decreased the concentration of leaf fatty acid and protein by 26–31 and 5.0–6.0%, while it had no effects on the concentrations of leaf free amino acid (Figures 5A–E). However, changes in the accumulations of these leaf quality variables did not correspond to changes in their concentrations as affected by CO_2_ elevation. Compared with other two CO_2_ treatments, the accumulations of DeCO_2_ significantly increased leaf fatty acid, starch, soluble sugar, total free amino acid, and protein by 8–171% (Figures 5F–J). In general, significantly higher differences among CO_2_ treatments are ranked as DeCO_2_ > (D + N)eCO_2_ ≥ NeCO_2_ > ACO_2_ for leaf starch and soluble sugar content, asDeCO_2_ > ACO_2_ ≈ (D + N)eCO_2_ ≈ NeCO_2_ for leaf fatty acid content, and as DeCO_2_ > (D + N)eCO_2_ > NeCO_2_ ≈ ACO_2_ for leaf total free amino acid and protein content (Figures 5F–J).

## Discussion

### Carbohydrate Accumulations

Carbohydrates in mulberry leaves are the major energy source for *Bombyx mori* L. (Horie, 1978). In the present study, leaf soluble sugar and starch in mulberry “Gui-sang-you 62” were, respectively, increased by 16 and 34% under 710/760 μmol mol^–1^ eCO_2_ (Figures 5F,G). Sekhar et al. (2014, 2015) found that 550/550 μmol mol^–1^ eCO_2_ triggered a remarkable increase in leaf total sugar in the mulberry genotypes of S13 and K2 by 23 and 19%, and starch by 30 and 35%, respectively. In addition, compared to S13, K2 had more total soluble sugars (12%) and starch (25%). These results showed that the effects of eCO_2_ varied from plant species to species. As a substrate for plant photosynthesis, eCO_2_ certainly affects the CO_2_ assimilation processes by increasing intercellular CO_2_ and carboxylation efficiency of Rubisco while reducing stomatal conductance and photorespiration (Sekhar et al., 2014, 2015; Li et al., 2017; Liu et al., 2019), leading to an accumulation of soluble sugar and starch. Interestingly, significant differences in the accumulation of carbohydrates were found in mulberry grown under altered daytime vs. nighttime, and continuous eCO_2_ treatment (Figures 5F,G). With low daytime CO_2_ and photosynthesis, NeCO_2_ could repress functionally important respiration and thus have important consequences for plant growth and yield production (Figure 2A; Bunce, 2002, 2014). When compared with soybean grown under 250/1,000 μmol mol^–1^ in NeCO_2_, their respiration was reduced, while photosynthesis was increased when grown under 1,000/1,000 μmol mol^–1^ (D + N)eCO_2_, resulting in an increased plant growth (Griffin et al., 1999). However, 710/760 μmol mol^–1^ (D + N)eCO_2_ did not result in such a large increase in mulberry biomass production and carbohydrate accumulation as these did under 710/460 μmol mol^–1^ DeCO_2_ (Figures 2, 5F,G). These results remain somewhat controversial with other previous outcomes from different plant species. For example, greater biomass in *Acer rubrum* and *Glycine max* but less biomass in *Amaranthus retroflexus* and *Medicago sativa* were observed when grown under 700/700 μmol mol^–1^ (D + N)eCO_2_ compared with under 700/350 μmol mol^–1^ DeCO_2_ (Bunce, 2003). *Xanthium strumarium* grown under 900/900 μmol mol^–1^ (D + N)eCO_2_ were greater than those grown under 900/350 μmol mol^–1^ DeCO_2_ (Reuveni et al., 1997). In contrast, total plant biomass production was similar, though seed yield was greater, when soybean was grown under 1,000/1,000 μmol mol^–1^ (D + N)eCO_2_ compared with under 1,000/250 μmol mol^–1^ DeCO_2_ (Griffin et al., 1999). Therefore, these varied results demonstrated that responses of plant performance to different daytime and/or nighttime eCO_2_ modulations are plant species dependent. With regard to the data presented in this paper, it seemed that the reduced respiration under high CO_2_ at nighttime had led to a reduced supply of energy-rich compounds including ATP and NADH (Bunce, 2001; Asensio et al., 2015), which could affect the energy required for N assimilation (Bloom et al., 2010; Asensio et al., 2015; Jauregui et al., 2015).

### Accumulations of Nitrogen Compounds

Plant carbohydrate concentrations can influence the concentrations of other plant constituents such as nitrogen compounds and mineral content. Mulberry varieties possessing higher leaf N, amino acid, and protein are nutritiously superior to the growth and development of silkworm (Machii and Katagiri, 1991; Sujathamma and Dandin, 2000). Our results showed that eCO_2_ in daytime and/or nighttime decreased leaf N and protein concentrations (Figures 3F, 5I,J). The decline in N concentration could be caused by an inhibition of leaf N assimilation (Tcherkez et al., 2020). Jauregui et al. (2015) found that a combination of elevated temperature (4°C) and 700 μmol mol^–1^ CO_2_ inhibited the activity of leaf nitrate (NO_3_^–^) reductase and glutamine synthetase, leading to a decreased synthesis of leaf total amino acid and soluble protein in wheat. Nighttime eCO_2_ also decreased both NO_3_^–^ assimilation and dark respiration in plants that relied on NO_3_^–^ (Asensio et al., 2015). The inhibited nighttime respiration, carbohydrate translocation, and NO_3_^–^ assimilation most likely explain the slower growth of plants exposed to eCO_2_ at night (Bunce, 2003; Asensio et al., 2015). Studies had found that the inhibition of leaf N assimilation under eCO_2_ is because of the reduction in photorespiration rates (Asensio et al., 2015; Jauregui et al., 2015). eCO_2_ decreases photorespiration and also the availability of NADH in the cytoplasm and thereby decreases the amount of reductant available for NO_3_^–^ reduction (Bloom et al., 2002, 2010, 2012). The mechanism may also involve processes in the chloroplast stroma that competes for reduced ferredoxin since NO_3_^–^ assimilation goes ahead only if the availability of reduced ferredoxin exceeds that which is required for the NADPH formation (Backhausen et al., 2000). Indeed, there was a decrease of 24–67% in leaf Fe concentration when plants were grown under eCO_2_ compared with that grown under ACO_2_ (Figure 3), while Fe is essential for a variety of ferredoxin relevant enzymes during the photosynthesis and N assimilation processes. Despite the decline in leaf N, amino acid, and protein concentrations, eCO_2_ did not markedly disturb the balance of C and N metabolism in plants (Figures 3A, 5D,E). Significantly positive linear relationships between leaf N, amino acid or protein accumulation, and biomass production also suggested that an increased plant biomass was associated with an enhanced plant N uptake and accumulation (Supplementary Figures S2B, S3D,E, *R*^2^ = 0.91–0.94, *P* < 0.001).

### Accumulations of Mineral Elements

It is documented that plant responses to eCO_2_ have led to stomatal closure, which directly affects transpiration rates and therefore slows down the mass flow of nutrients (Taub and Wang, 2008; Houshmandfar et al., 2015). Soybeans under eCO_2_ did have a significantly lower daytime stomatal conductance than those under ACO_2_ (Griffin et al., 1999). The decline in leaf N concentration exposed to eCO_2_ (Figure 3) could therefore be due to such a decreased N transport from belowground to aboveground. Our results showed that either DeCO_2_ (710/460 μmol mol^–1^), NeCO_2_ (410/760 μmol mol^–1^), or (D + N)eCO_2_ (710/760 μmol mol^–1^) decreased the concentrations of leaf P, Mg, Fe, Cu, and Zn, but the reduction is the least in NeCO_2_ (Figure 3). Similar depletions in the nutritional status under eCO_2_ have been reported in a number of woody species including *Quercus serrata* and *Quercus mongolica* (Shi et al., 2016), *Coffea arabica* (Martins et al., 2014), Norway spruce (Marshall and Linder, 2013), and *Larix kaempferi* (Shinano et al., 2007). Such declines are very relevant to the plant physiological processes. Mg is essential for the formation of light-harvesting chlorophyll a/b complexes and an indicator of leaf greenness (Shaul, 2002; Teklic et al., 2009). Like the result in Figure 3, a lower leaf Mg was associated with a reduced chlorophyll in two mulberry genotypes (K2 and S13) under 550 μmol mol^–1^ eCO_2_ (Sekhar et al., 2014). In terms of Rubisco and chlorophyll, a reduction in Mg content under eCO_2_ has been proposed as a key response that underlines the importance of transpiration rates and xylem flux (Houshmandfar et al., 2015). More than half of cellular Fe and Cu are found in chloroplasts and participate in photosynthesis, mitochondrial respiration, and N metabolism (Hänsch and Mendel, 2009). As a component of enzymes for protein synthesis, a reduction in grain protein concentration of wheat was well correlated with Fe (*R*^2^ = 0.70) and Zn (*R*^2^ = 0.50) (Fernando et al., 2012). Overall, in this study, we found significant differences in leaf yield and harvest of nutritious compounds of mulberry grown under eCO_2_ at 710/460 μmol mol^–1^ DeCO_2_, 410/760 μmol mol^–1^ NeCO_2_, and 710/760 μmol mol^–1^ (D + N)eCO_2_, compared with plants grown under ACO_2_. We speculated that limited energy levels resulting from a reduced respiratory under NeCO_2_ could slow plant growth. When compared to DeCO_2_, (D + N)eCO_2_ reduced the concentrations of leaf Mg, Cu, and Zn, which associate to plant photosynthesis, mitochondrial respiration, and N metabolism. As a result, lower mulberry leaf yield and harvest of nutritious compounds were displayed under (D + N)eCO_2_ than under DeCO_2_, leading to a consequent poor leaf quantity and quality for silkworm.

## Conclusion

This study showed that a positive response of mulberry growth and leaf quality to eCO_2_ was greater at daytime than at nighttime. Compared to ACO_2_, the amounts of leaf N, P, K, Ca, Mg, Mn, B, Cu, Fe, and Zn were significantly increased under either DeCO_2_ or (D + N)eCO_2_, except leaf Fe under NeCO_2_. Our results also showed that DeCO_2_ and (D + N)eCO_2_, not NeCO_2_, increased biomass production and thus the harvest of nutrients and accumulations of leaf carbohydrates (starch, soluble sugar, and fatty acid) and N-containing compounds (free amino acid and protein), though there were some decreases in the concentration of leaf N, P, Mg, Fe, and Zn. However, (D + N)eCO_2_ did not result in as large an increase in mulberry leaf yield and harvest of nutritious compounds for silkworm as did DeCO_2_. Although no significant effects on yield were observed under NeCO_2_, there was a positive effect on nutritional quality on leaf to an extent. In conclusion, a positive effect of eCO_2_ on plant biomass production would require an increased nutrient uptake for the synthesis of leaf carbohydrate fatty acid and N-containing compound accumulation. Higher nutrient demand is ranked as DeCO_2_ > (D + N)eCO_2_ > NeCO_2_. The depletion of leaf N, P, Mg, Fe, and Zn, and fatty acid, amino acid, and protein concentrations in mulberry leaves under eCO_2_ would certainly affect the growth and thus the quality and quantity of cocoons. Therefore, a rational input of external essential elements, particularly N, P, Fe, Mg, and Zn is vital to meet the nutritional requirements of mulberry trees under future CO_2_ elevation.

## Data Availability Statement

The original contributions presented in the study are included in the article/Supplementary Material, further inquiries can be directed to the corresponding author/s.

## Author Contributions

XX and XH conceived and designed the experiments. SS, YQ, MW, XD, and CX performed the pot experiments and collected the samples. SS, YQ, and MW were responsible for the determination of the leaf quality parameters. XH, SS, and XX provided fund support. XD analyzed the mineral element data with the help of CX. SS and YQ wrote the manuscript. All authors have read and approved the final manuscript.

## Conflict of Interest

The authors declare that the research was conducted in the absence of any commercial or financial relationships that could be construed as a potential conflict of interest.

## Footnotes

1 https://www.co2.earth
