# Supplementary material for: Daytime, Not Nighttime, Elevated Atmospheric Carbon Dioxide Exposure Improves Plant Growth and Leaf Quality of Mulberry (Morus alba L.) Seedlings
Source: Front Plant Sci. 2021 Feb 4;11:609031. doi: 10.3389/fpls.2020.609031 (PMC7890035; doi:10.3389/fpls.2020.609031)
Supplement: Supplementary file 1 [file Data_Sheet_1.docx]

**Supplementary material**

**Daytime, not nighttime, elevated atmospheric CO_2_exposure improves plant growth and leaf quality of mulberry(*Morusalba*L.)seedlings**

Songmei Shi^a,✝^, YulingQiu^a,✝^, Miao Wen^a^, Xiao Xu^b^, Xingshui Dong^a^, ChenyangXu^a^, Xinhua He^a, c*^

^a^Centre of Excellence for Soil Biology, School of Resource and Environment, Southwest University, Chongqing 400715, China

^b^Key Laboratory of Southwest China Wildlife Resources Conservation (China West Normal University), Ministry of Education, Nanchong 637009, Sichuan, China

^c^School of Biological Sciences, University of Western Australia, Perth, WA 6009, Australia

^✝^These authors contributed equally to this work

*Correspondences: xinhua.he@uwa.edu.au

**Figure captions**

**Fig.S1**Overview of environmentally controlled growth chambers

A total of 12 growth environmentally controlled glass-made chamberswere constructed in 2016 at the National Monitoring Station of SoilFertility and Fertilizer Efficiency on Purple Soils, which is located on the Southwest University Campus, theBeibei District (29°48′N, 106°24′E, 266.3 m above the sea level), Chongqing, China. The temperatures and humidity inside the growth chambers were also automatically maintained as the same as those outside the chambers. The temperature, humidity, light and CO_2_ concentration had been monitored every 6 second by a CO_2_auto-controlling facility. The plants grown in pots inside chambers had the same growth conditions (nitrogen-fertilization, temperature, light intensity, etc.), except CO_2_ concentrations: ambient CO_2_ (ACO_2_, 410µmol mol^-1^ daytime/460µmol mol^-1^nighttime), daytime eCO_2_only (DeCO_2_, 710/460µmol mol^-1^), nighttime eCO_2_only (NeCO_2_, 410/760µmol mol^-1^), and continuousdaytime/nighttime eCO_2_(D+NeCO_2_, 710/760µmol mol^-1^).

**Fig. S2**Relationships between leaf biomass production and leaf carbon (C), nitrogen (N), phosphorous (P), potassium (K), calcium (Ca), magnesium (Mg), Manganese (Mn), boron (B), copper (Cu), iron (Fe) and zinc (Zn) accumulation in mulberry plants. Data are means ± SD, *n* = 12.See treatment abbreviations in Fig.S1.

**Fig. S3**Relationships between leaf soluble sugar, starch, fatty acid, total free amino acid and proteinaccumulation with leaf biomass productionin mulberry plants. *n*=12.See treatment abbreviations as in Fig.S2.

**Fig. S1**


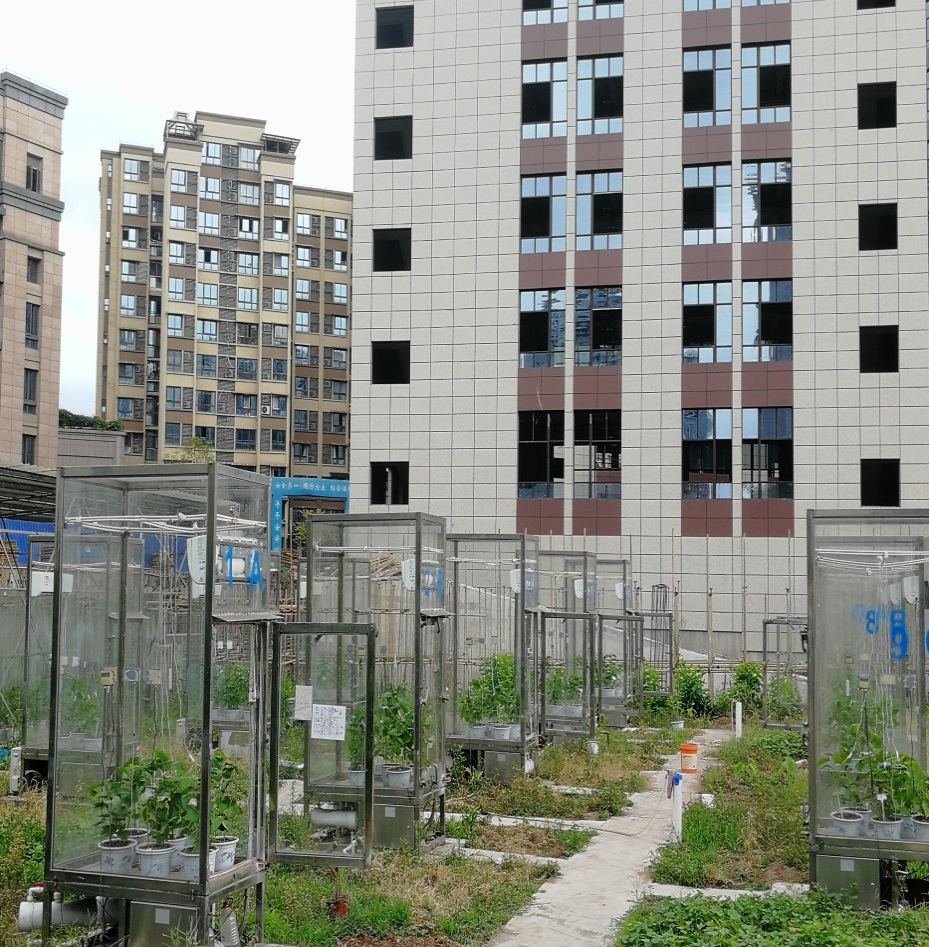


**Fig. S2**

**Fig.S3**
